# Efficacy of Nutritional Interventions in Adult Poststroke Rehabilitation: Protocol for a Systematic Review and Trial Sequential Meta-Analysis

**DOI:** 10.2196/79478

**Published:** 2026-03-24

**Authors:** Chen Chen, Su-xiang Zhang, Shao-hua Chen, Yan Cao, Ji-ming Tao

**Affiliations:** 1 Department of Rehabilitation Medicine, Shuguang Hospital Affiliated to Shanghai University of Traditional Chinese Medicine, 185 Pu 'an Road, Huangpu District, Shanghai, 200001, China, 86 13386192862

**Keywords:** stroke, nutrition, systematic review, rehabilitation, randomized controlled trial

## Abstract

**Background:**

Malnutrition is a prevalent and serious concern in adult survivors of stroke, often worsening their clinical status and leading to a poor prognosis. However, the effectiveness of nutritional interventions in this population remains uncertain. Furthermore, the impact of nutritional support on functional recovery, especially in relation to rehabilitation outcomes, has not been sufficiently investigated.

**Objective:**

This meta-analysis aims to synthesize evidence from existing randomized controlled trials (RCTs) to determine whether nutrient supplements confer measurable benefits in poststroke rehabilitation, with overall mortality as the primary outcome and multiple relevant indicators as secondary outcomes.

**Methods:**

RCTs that compare nutrient supplements and a standard diet or placebo supplements in adult survivors of stroke will be included. Literature searches will be conducted in the PubMed, Web of Science, Embase, China National Knowledge Infrastructure, Wanfang, and Cochrane Library databases. Two reviewers will independently perform the processes of literature retrieval, screening, data extraction, and assessment of risk of bias. Risk of bias in included studies will be evaluated using the revised Cochrane risk-of-bias tool for RCTs. Review Manager will be used for data pooling. Subgroup analysis, trial sequential analysis, and sensitivity analysis will be conducted.

**Results:**

This study was funded in 2023 (2023ZDFC0301) and registered in PROSPERO (CRD420251028426) prior to initiation. As of November 25, 2025, literature screening has not yet started. The planned timeline is as follows: complete literature screening by February 28, 2026; finalize full-text assessment and data extraction by May 30, 2026; conclude trial sequential analysis by June 30, 2026; and complete manuscript preparation for publication by July 30, 2026.

**Conclusions:**

This systematic review and meta-analysis will provide evidence for the efficacy of nutritional interventions in adult poststroke rehabilitation, which may inform clinical guidelines and optimize rehabilitation strategies for this vulnerable population.

## Introduction

Stroke remains a leading cause of long-term disability and mortality worldwide, with survivors often facing significant physical and cognitive impairments that hinder functional recovery. Poststroke rehabilitation is crucial for improving patient outcomes, yet recovery can be complicated by malnutrition, muscle wasting, and secondary infections, which further exacerbate disability and increase mortality risk [1-6]. Nutritional deficiencies, particularly in protein and essential micronutrients, are common among survivors of stroke due to dysphagia, reduced appetite, and metabolic stress, potentially delaying recovery and worsening neurological deficits [7-12]. Given these challenges, nutrient supplementation has emerged as a promising adjuvant therapy to enhance rehabilitation by addressing malnutrition, supporting muscle synthesis, and improving immune function.

Current evidence suggests that nutrient supplements—including proteins, amino acids, vitamins, and minerals—may positively influence stroke recovery by mitigating catabolism, enhancing neurological repair, and reducing infection rates [13,14]. However, findings across randomized controlled trials (RCTs) remain inconsistent, with variations in supplement type, dosage, and patient populations leading to conflicting results [15,16]. While some studies report significant improvements in muscle strength, mobility, and biochemical markers, others show negligible effects on mortality or functional outcomes [17-22]. These discrepancies highlight the need for a comprehensive meta-analysis to evaluate the efficacy of nutrient supplementation in adult survivors of stroke, particularly in comparison with standard dietary care or placebo.

This meta-analysis aims to synthesize evidence from existing RCTs to determine whether nutrient supplements confer measurable benefits in poststroke rehabilitation. The primary outcomes include overall mortality, while the secondary outcomes encompass infection rates; neurological function (National Institutes of Health Stroke Scale [NIHSS] score); nutritional status (albumin, hemoglobin, and protein intake); and physical performance (handgrip strength and 6-minute walking distance). By clarifying the role of nutritional interventions in stroke recovery, this study may inform clinical guidelines and optimize rehabilitation strategies for this vulnerable population.

## Methods

### Protocol and Registration

In adherence to the PRISMA-P (Preferred Reporting Items for Systematic Reviews and Meta-Analyses Protocols) guidelines, we meticulously reported this systematic review and meta-analysis. Prior to initiation, the protocol for this comprehensive study was formally registered in the PROSPERO database, ensuring transparency and rigor in our research methodology [23] (registration ID CRD420251028426). This protocol describes a future systematic review. As of November 25, 2025, no screening or data extraction has been initiated and no results exist. The team will complete literature screening by February 28, 2026; finalize full-text assessment and data extraction by May 30, 2026; conclude trial sequential analysis by June 30, 2026; and complete manuscript preparation by July 30, 2026.

### Inclusion Criteria

#### Participants

Participants include adults (aged ≥18 years) with confirmed stroke (including ischemic and hemorrhagic stroke diagnosed via computed tomography or magnetic resonance imaging). Stroke severity is not restricted (mild to severe; NIHSS score 0‐42). Patients with or without poststroke complications (eg, dysphagia, muscle weakness, and infections) are included, except for those meeting the exclusion criteria.

#### Intervention

The intervention of interest is defined as nutritional supplements, which refer to oral, enteral, or parenteral preparations containing one or more nutrients (eg, protein, amino acids, vitamins, minerals, and fatty acids) designed to supplement dietary intake. To be included, these supplements must be administered for a minimum of 2 weeks and a maximum of 6 months. It is important to note that this definition specifically encompasses nutrient supplements, while broader dietary intervention types (eg, high-protein diets and the Mediterranean diet) are excluded. For comparison, the control group comprises patients who receive only a standard diet or placebo supplements.

#### Primary Outcome

The primary outcome is overall mortality, defined as all-cause mortality during the study follow-up period. The primary time point for assessment is 3 months after the initiation of the nutritional intervention; if multiple follow-up time points are available (eg, 6 months or 1 year), data at the longest available follow-up will be prioritized.

#### Secondary Outcomes

Secondary outcomes are described below.

The incidence of overall infection is defined as the occurrence of any infection (eg, pneumonia, urinary tract infection, and sepsis) during the intervention period. The assessment time point will be throughout the nutritional intervention period (minimum 2 weeks and maximum 6 months).

The NIHSS is a validated tool for measuring neurological deficit severity (score range: 0‐42, higher scores indicate more severe deficits). Acceptable measurement tools are the original NIHSS or standardized translated versions. A clinically meaningful change will be a reduction of ≥4 points. The assessment time point will be at baseline and after the intervention (3 months).

Albumin (g/L) will be measured via venous blood sampling. A clinically meaningful change will be an increase of ≥3 g/L from baseline. The assessment time point will be at baseline and after the intervention (3 months).

Hemoglobin (g/L) will be measured via venous blood sampling. A clinically meaningful change will be an increase of ≥10 g/L from baseline. The assessment time point will be at baseline and after the intervention (3 months).

Daily protein intake (g/d) will be assessed via 24-hour dietary recall, food frequency questionnaire, or dietary records. Acceptable measurement tools are validated dietary assessment instruments. The assessment time point will be at baseline and after the intervention (3 months).

Handgrip strength (kg) will be measured using a dynamometer (eg, Jamar dynamometer) with the dominant hand, following standard operating procedures (patient seated, elbow flexed at 90°, and forearm neutral). A clinically meaningful change is an increase of ≥3 kg. The assessment time point will be at baseline and after the intervention (3 months).

The 6-minute walking test (m) is measured as the maximum distance walked in 6 minutes on a flat surface, following American Thoracic Society guidelines. A clinically meaningful change is an increase of ≥54 m. The assessment time point will be at baseline and after the intervention (3 months).

### Study Design

We will include RCTs. Any other literature, including nonrandomized clinical controlled trials, retrospective research studies, conference abstracts, case reports, duplicate publications, and literature without data will not be considered.

### Eligibility Criteria

Inclusion and exclusion criteria are described in Textbox 1. Crossover studies with an adequate washout period (≥2 weeks) and separable data will be included and analyzed using the generic inverse variance model.

Textbox 1. **Inclusion and exclusion criteria.**

Inclusion criteria

- Adults (aged ≥18 years) with confirmed stroke (ischemic or hemorrhagic) diagnosed via computed tomography or magnetic resonance imaging
- Stroke severity of any grade (National Institutes of Health Stroke Scale score 0-42)
- Patients with or without poststroke complications (eg, dysphagia, muscle weakness, and infections)
- Nutritional supplement intervention (oral, enteral, or parenteral) containing one or more nutrients, administered for 2 weeks to 6 months
- Control group receiving standard diet or placebo supplements
- Randomized controlled trial study design
- Outcome data including at least one predefined primary or secondary outcome

Exclusion criteria

- Pregnant or lactating women
- Patients with cancer (due to metabolic disturbances and confounding from anticancer treatments)
- Participation in other clinical studies within 3 months before the study
- Complete aphasia with inability to complete outcome assessments and persistent unconsciousness (Glasgow Coma Scale score <8 for >72 hours after stroke)
- Patients who refuse nutritional intervention or follow-up
- Crossover studies with washout period <2 weeks (insufficient to eliminate residual effects of the first intervention) or for which outcome data cannot be separated by the intervention period

### Literature Sources and Retrieval Strategy

To ensure accuracy and completeness, 2 reviewers (CC and YC) will independently conduct a comprehensive search of articles across 7 databases and 1 clinical trial registry: PubMed, Web of Science, Embase, China National Knowledge Infrastructure, Wanfang, Chinese Biomedical Literature Database (SinoMed), the Cochrane Library, and the International Clinical Trials Registry Platform. The International Clinical Trials Registry Platform search will focus on completed but unpublished RCTs related to nutritional interventions in poststroke rehabilitation. The detailed retrieval strategy (combining Medical Subject Headings terms and free text keywords) is outlined in Table 1.

**Table 1.** The detailed retrieval strategy (combining Medical Subject Headings [MeSH] terms and free text keywords).

Step	Search term (MeSH+free text)	Boolean operation
1	Stroke [MeSH] OR cerebrovascular apoplexy OR intracranial arteriosclerosis OR intracranial embolism OR brain infarction OR cerebral infarction OR cerebral hemorrhage OR ischaemic stroke OR haemorrhagic stroke	OR
2	Nutritional supplements [MeSH] OR nutrition OR energy OR calorie OR carbohydrate OR protein OR amino acid OR fat OR fatty acid OR vitamin OR mineral OR branched-chain amino acid OR whey protein OR omega-3 fatty acid	OR
3	Rehabilitation [MeSH] OR post-stroke rehabilitation OR functional recovery OR muscle strength	OR
4	Step 1 AND Step 2 AND Step 3	AND
5	Filter: randomized controlled trial, humans, and adults (aged ≥18 years)	—^a^

a Not applicable.

### Literature Screening and Data Extraction

To ensure meticulous accuracy and comprehensive coverage, 2 reviewers (CC and YC) will adhere to the PRISMA (Preferred Reporting Items for Systematic Reviews and Meta-Analyses) guidelines for rigorous literature screening and data extraction. The screening process will proceed in 2 stages. First, reviewers will independently screen titles and abstracts of retrieved records against the inclusion and exclusion criteria. Records deemed potentially eligible or uncertain will be retained for full-text assessment. Second, reviewers will independently assess the full text of retained records to confirm eligibility. Any discrepancies at either stage will be resolved through discussion; if consensus cannot be reached, a third senior reviewer (JT) will be consulted. The extracted data will include study design, baseline patient information, and statistics on overall mortality, incidence of overall infection, NIHSS score, albumin (g/L), hemoglobin (g/L), daily protein intake (g/d), handgrip strength (kg), and 6-minute walking test (m).

### Assessment of Risk of Bias

To ensure accuracy and completeness, 2 reviewers (CC and SC) will independently evaluate the risk of bias in the included studies. They will use the Revised Cochrane risk-of-bias tool (ROB 2) to assess the potential bias in RCTs. Their evaluation will encompass 5 critical dimensions that could impact study quality: bias related to the randomization process, deviations from the intended interventions, missing outcome data, measurement of outcomes, and the selection of reported results. Any discrepancies will be resolved through established protocols.

### Ethical Considerations

No ethics approval is required for this review. Our findings will be submitted to peer-reviewed journals.

### Statistical Analysis

The Review Manager software (version 5.4; Cochrane Informatics & Technology Services) will be used to aggregate the data and produce forest plots. For dichotomous variables, risk ratios and 95% CIs will be calculated and analyzed using the Mantel-Haenszel method. Continuous variables will be pooled and analyzed as the mean difference with 95% CIs, using the inverse variance method. The statistical significance level (α) is set at .05. Statistical differences are deemed significant if the *P* value <.05. A random effects model will be adopted a priori for all meta-analyses, considering expected clinical heterogeneity (eg, variations in supplement types, doses, durations, and patient stroke types) regardless of *I*² values. The *I*² statistic will be used to quantify statistical heterogeneity: <25% indicates low heterogeneity, 25% to 50% indicates moderate heterogeneity, and >50% indicates high heterogeneity. Publication bias will be assessed only when the number of included studies exceeds 10, as a small number of studies could compromise the robustness of the tests. For missing outcome data, we will first contact the original study authors; if unavailable, we will use multiple imputation or conduct a sensitivity analysis excluding studies with excessive missing data (>20%). To ensure the reproducibility of statistical analyses, we specify the following operational rules: MD will be the primary effect measure for continuous outcomes where all included studies report the same outcome using identical, clinically interpretable units (eg, albumin in g/L, hemoglobin in g/L, handgrip strength in kg, and 6-minute walking distance in m), while standardized mean difference (SMD; Hedges *g* and corrected for small sample size bias) will be used only when studies report the same core outcome using different measurement units or noncomparable validated scales, with SMD effect sizes interpreted per Cohen guidelines (0.2=small effect, 0.5=moderate effect, and 0.8=large effect); for continuous outcomes with convertible nonidentical units, raw data will be uniformly converted to the International System of Units before mean difference calculation, with conversion factors reported and verified by 2 independent reviewers, and SMD will be the sole effect measure for outcomes with nonconvertible units or different measurement scales. For cluster randomized trials, only those with available individual participant data or an adjusted effective sample size accounting for the intracluster correlation coefficient (ICC) will be included, and a conservative ICC of 0.05 (per Cochrane Handbook recommendations) will be used to calculate the effective sample size if the ICC is not reported in the original study, while cluster randomized trials with no available individual participant data, ICC, or raw group-level data will be excluded from the primary meta-analysis and included in a separate sensitivity analysis if raw group-level data are available. For crossover trials meeting the inclusion criteria (washout period ≥2 weeks, separable outcome data by intervention period), only data from the first treatment period will be used for the primary meta-analysis to eliminate carryover effects and avoid double counting of participants, and separable outcome data from the second treatment period (if available) will be used in a prespecified sensitivity analysis to verify the robustness of the primary results. All statistical processing steps, including unit conversion, effect measure selection, and trial design adjustments, will be fully and transparently reported in the final manuscript to ensure complete reproducibility.

### Subgroup Analysis and Sensitivity Analysis

Subgroup analyses will be performed based on the following variables to explore potential effect modifiers: participant characteristics, including age (<65 years vs ≥65 years), stroke type (ischemic vs hemorrhagic), diabetes status (with vs without diabetes mellitus), and stroke severity (mild to moderate [NIHSS ≤16] vs severe [NIHSS >16]). Sensitivity analyses for the primary outcome (overall mortality) will include the following methods: (1) excluding studies with a high risk of bias (ROB 2 assessment), (2) excluding studies with a small sample size (<50 participants), (3) excluding studies with excessive missing data (>20%), (4) using a fixed effects model instead of a random effects model to assess the impact of model selection on results, and (5) excluding studies with follow-up duration of less than 6 months to assess the impact of follow-up length on results.

### Trial Sequential Analysis

Random errors can lead to spurious conclusions when a meta-analysis involves a limited number of trials and a small patient population. Trial sequential analysis (TSA) will be used to mitigate the risks associated with random errors due to insufficient sample size or repeated testing and to estimate the required information size for the meta-analysis. We will conduct TSA using the TSA software (version 0.9.5.10 beta). The type I error rate and power will be set at 5% and 80%, respectively.

### Certainty of Evidence

Certainty of the evidence will be assessed using GRADEpro, a tool developed by the Grading of Recommendations Assessment, Development, and Evaluation (GRADE) Working Group [24]. The assessment criteria for certainty include, but are not limited to, the initial study design, risk of bias, imprecision, indirectness, and inconsistency. Following the guidelines, the certainty of the evidence will be categorized as “high,” “moderate,” “low,” or “very low” with the assistance of GRADEpro.

### Patient and Public Involvement

Patients and/or the public were not involved in the design, conduct, reporting, or dissemination plans of this research.

## Results

This study was funded in 2023 (2023ZDFC0301) and registered in PROSPERO (CRD420251028426) prior to initiation. As of November 25, 2025, literature screening has not yet started. The planned timeline is as follows: complete literature screening by February 28, 2026; finalize full-text assessment and data extraction by May 30, 2026; conclude TSA by June 30, 2026; and complete manuscript preparation for publication by July 30, 2026.

**Figure 1.**
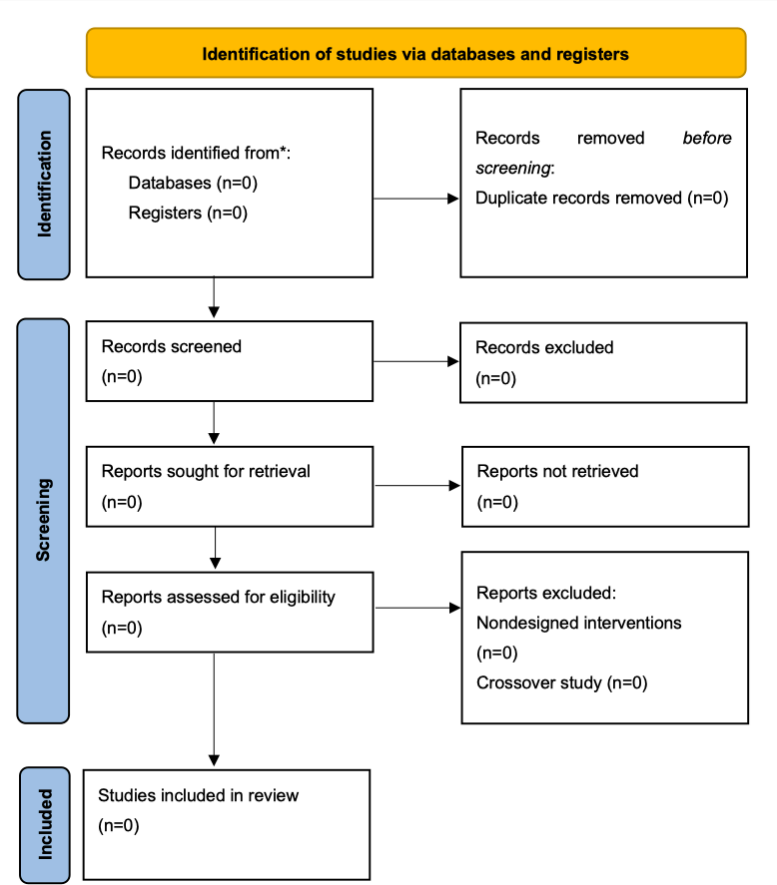
PRISMA (Preferred Reporting Items for Systematic Reviews and Meta-Analyses) flowchart of the study inclusion process.

The flowchart of the study selection process is illustrated in Figure 1.

## Discussion

### Anticipated Findings

This meta-analysis adheres to PRISMA-P guidelines and a preregistered protocol, using rigorous methods to assess nutrient supplementation’s role in stroke rehabilitation. A comprehensive search across multiple databases and robust statistical analyses, including trial sequential analysis, will be implemented to ensure reliability. The protocol is specifically designed to minimize bias while maximizing the clinical relevance of our findings for stroke rehabilitation practice.

The study design presents several notable strengths that merit discussion. Our exclusive focus on RCTs provides the highest level of evidence for evaluating intervention efficacy, while the broad inclusion criteria encompassing various nutrient supplements increase the generalizability of findings across different clinical settings. The comprehensive outcome assessment, ranging from clinical end points (mortality) to functional measures (6-minute walk test) and biochemical markers (albumin levels), allows for a multidimensional evaluation of nutritional interventions. Furthermore, the planned subgroup analyses by age and sensitivity analyses enhance our ability to detect potential effect modifiers and verify the robustness of the results. The incorporation of GRADE methodology for evidence certainty assessment adds further credibility to our conclusions.

### Strengths and Limitations of This Study

This systematic review and meta-analysis follows a rigorous methodology with strict inclusion criteria. The use of ROB 2 ensures a rigorous evaluation of study quality. The certainty of the evidence may be limited by heterogeneity in study quality and the small sample sizes of the included trials.

Several methodological considerations also warrant attention. First, while our inclusion of only RCTs enhances internal validity, it may limit generalizability to real-world medical conditions where patient populations are more heterogeneous. The variability in supplement types (eg, proteins and amino acids) and administration protocols across studies introduces clinical heterogeneity that could obscure true treatment effects. Our protocol attempts to address this through subgroup analyses, but these may be limited by the available data. Second, the focus on short-term outcomes (eg, 6-minute walk test and albumin levels) leaves open questions about sustained benefits—a critical gap given stroke’s chronic nature. The nutritional field particularly faces measurement challenges; for instance, protein intake reporting often relies on recall methods vulnerable to bias. Third, while trial sequential analysis will be performed to guard against random errors, its utility depends on sufficient sample sizes that may be unrealistic in this research area. These limitations highlight an urgent need for standardized, large-scale trials with longer follow-up periods and objective nutritional biomarkers.

### Conclusions and Future Research

Future research should prioritize 3 key directions. Mechanistic studies are needed to clarify how specific nutrients (eg, branched-chain amino acids) influence neuroplasticity and muscle synthesis at the molecular levels, which could explain current inconsistent findings. More trials that compare different supplementation strategies (eg, timing relative to stroke onset and combination with exercise) would help optimize clinical protocols. Most importantly, the field requires the development of validated, stroke-specific nutritional assessment tools—current measures such as handgrip strength and serum markers may not fully capture rehabilitation progress. Emerging technologies such as continuous glucose monitoring or wearable sensors could provide more dynamic nutritional data. There is also growing recognition that stroke recovery may benefit from personalized nutrition approaches based on genetic polymorphisms affecting nutrient metabolism. Finally, integrating nutritional interventions with other rehabilitation modalities represents a promising but underexplored avenue that could yield greater benefits for functional recovery. Addressing these gaps through collaborative, multidisciplinary research will be essential for advancing evidence-based nutritional strategies in stroke care.

## Abbreviations

GRADE: grading of recommendations assessment, development, and evaluation
ICC: intracluster correlation coefficient
NIHSS: National Institutes of Health Stroke Scale
PRISMA: Preferred Reporting Items for Systematic Reviews and Meta-Analyses
PRISMA-P: Preferred Reporting Items for Systematic Reviews and Meta-Analyses Protocols
RCT: randomized controlled trial
ROB 2: Revised Cochrane risk-of-bias tool
SMD: standardized mean difference
TSA: trial sequential analysis
